# Momentary engagement in simultaneous versus consecutive interpreting: through the lens of translanguaging and CDST

**DOI:** 10.3389/fpsyg.2023.1180379

**Published:** 2023-05-05

**Authors:** Lili Han, Jing Lu, Zhisheng (Edward) Wen, Yuan Tian

**Affiliations:** ^1^Faculty of Languages and Translation, Macao Polytechnic University, Macau, Macao SAR, China; ^2^English Language and Literature, Hong Kong Shue Yan University, North Point, Hong Kong SAR, China

**Keywords:** simultaneous interpreting, consecutive interpreting, translanguaging moments, moment analysis, pause analysis, CDST, multicompetence

## Abstract

Through the lens of translanguaging theory and the complex, dynamic system theory (CDST) approach, the interpreting process is considered a highly complex and dynamic activity that engages the interpreter’s cognition, emotion, and action during successive “translanguaging moments” of meaning-making. Meanwhile, the two dominant types of interpreting, namely, simultaneous interpreting, and consecutive interpreting are assumed to entail distinct time sensitivity and consume different amounts of cognitive resources at different stages. Based on these assumptions, the present study analyzes interpreters’ momentary engagement during the distinct workflow tasks associated with these two modes of interpreting, with a view to probing their underlying non-linearity, self-organization and emergence dynamics from a micro-level perspective. Furthermore, we triangulated the textual description with multimodal transcription to portray these “translanguaging moments,” which are augmented with a follow-up emotional survey that corroborated our findings.

## 1. Introduction

Viewed from the lens of translanguaging (Li, 2011, 2018, 2022) and the complex dynamic (network) approach (Lin et al., 2021), the interpreting process can be regarded as a highly complex and dynamic cognitive activity that implicates a bi/multilingual individual (Aka the interpreter) engaging in successive “translanguaging moments” for meaning-making (Han et al., 2023). In this process, the interpreters’ multi-linguistic repertoires (e.g., L1, L2, and Lx proficiency) and their cognitive capacities (e.g., working memory, attentional control) constitute their *multicompetence* (Cook, 2016), which in turn interacts cooperatively or competitively with other internal or external factors to impact their interpreting performance. The composition and allocation of mental operations during the process of interpreting are best captured by the Effort Model (Gile, 1995/2009, 2016), in which simultaneous interpreting (SI) is conceptualized and demarcated as a process consisting of listening and analysis effort, short-term memory effort, speech production effort, and coordination effort, mathematically represented as L, M, P + C. In contrast, consecutive interpreting (CI) is composed of two phases, namely the comprehension phase and the reformulation phase (Gile, 1995/2009, 2016).

Although SI and CI are assumed to implicate different cognitive complexity (Xiao and Muñoz, 2020; Lin et al., 2021), it remains unknown whether they consume different amounts of cognitive resources and to what extent. As such, the present study sets out to analyze the momentary complexity and dynamic architecture of the interpreting process by focusing on the distinct workflow tasks of SI and CI, with a view to better understanding the underlying non-linearity, emergence and self-organization dynamics of these two interpreting modes. More importantly, we aim to advance current knowledge on interpreter engagement from a micro-level perspective as informed by the emerging theory of translanguaging in human cognition and communication (Li, 2011, 2018, 2022). Inspired by the key tenets and principles of translanguaging, which epitomize multilingual and multimodal resources, we combined textual description with multimodal transcription to analyze interpreters’ “translanguaging moments” during the SI and CI task performance. In particular, we video-taped and focused on the interpreters’ note-taking (specifically for CI), facial expressions, gestures, images, speech, and textual outputs during the interpreting process. Finally, we triangulated our findings with a follow-up emotional engagement survey (Dao et al., 2021).

## 2. Literature review

Recent scholarly developments in interpreting studies attest to the growing perception among researchers that, in understanding the processes of interpreting as the meaning-making practice, insights from such emerging concepts of multimodality, multisensory, and multisemiotics are making significant contributions. Under these views, interpreting is now conceived as a highly complex and dynamic cognitive activity engaging the interpreter in successive “moments” of meaning-making. During this process, s/he mobilizes a whole array of para-verbal resources, such as voice quality, cadence, inflection, rate of speech, and nonverbal resources, such as facial expressions, gestures, and gaze (Poyatos, 1997; Mason, 2001, 2009; Mason and Stewart, 2001; Pasquandrea, 2012; Pérez-González, 2014, 2020; Davitti, 2015; Davitti and Pasquandrea, 2017; Canals, 2021). As an integrated system of meaning-making, interpreting should thus be analyzed as a whole so that we can arrive at a thorough understanding of the communicative dynamics of the interlingual and intercultural transfer via the interpreter.

Meanwhile, in applied linguistics and language education, the past two decades have witnessed the emergence of the practical theory of translanguaging (Li, 2011, 2018, 2022), making its foray into a broad range of human cognition and communication domains that are characterized by superdiverse, multilingual, multicultural, and multisemiotic social contexts, including translation and interpreting practice (Baynham and Lee, 2019; Runcieman, 2021).

According to Li (2018), translanguaging refers to the “ability of multilingual speakers to shuttle between languages, treating the diverse linguistic resources that form their repertoire as an integrated system” (p. 10). Based on this, we adopt the working definition of translanguaging performance/spaces in the present study as the approach to language use that goes beyond the traditional boundaries of language and encourages speakers to use their full range of linguistic resources to communicate effectively. Translanguaging spaces are environments where this approach to language use is particularly evident, where individuals use a combination of languages, dialects, and registers to communicate and negotiate meaning. In interpreting contexts, translanguaging spaces refer to the moments when interpreters use their full linguistic repertoires to make meaning for themselves and for their clients, such as during conference interpreting when time constraints require interpreters to make instant albeit strategic decisions on how to convey meaning across languages.

Interpreting, when conceived as the translanguaging practice of meaning-making, is composed of multilayered “translanguaging spaces” (Li, 2011, 2018; Tai and Li, 2021) constantly and accumulatively created by dynamic interactional “moments” between the interpreter and the external environment within the broader social-cultural contexts as well as among the interpreter’s individual-based multicompetence blended with those multimodal affordances in the external environment (Han et al., 2023). In this multimodal, multisemiotic, and multisensory communicative process, the interpreters’ multi-linguistic repertoires (e.g., L1, L2, and Lx proficiency) and cognitive capacities (e.g., working memory, attentional control) constitute their “multicompetence” (Cook, 2016) interacting cooperatively and competitively in interpreting performance. Such a translanguaging lens for interpreting performance, when augmented by integrated research methods from neighboring disciplines such as the complex dynamic systems theory (CDST; Dong, 2018; Hiver and Al-Hoorie, 2020; Hiver et al., 2021) approach, allows us to adequately simulate, explain, and predict the emergence, self-organization, and real-time performance of interpreting. The engagement of individual students’ momentary involvement in interpreting is itself a complex and dynamic system comprised of emotion, motivation, mental action, and physical action in the ongoing task architecture (Symonds et al., 2020). This CDST insight sheds light on our analysis and understanding of the translanguaging moments in interpreting performance at a micro-level.

On the other hand, interpreting performance can be categorized and analyzed from different perspectives (Pöchhacker, 2015, pp. 228–29). For example, it can be classified in terms of modality, namely, we have spoken (language) interpreting versus signed (language) interpreting. Alternatively, it can be analyzed in terms of institutional contexts, which subsumes legal interpreting, healthcare interpreting, and educational interpreting. Nevertheless, it can be categorized in terms of the format of interaction; thus, we have dialog interpreting and conference interpreting. Most relevantly, in terms of the temporal relationship between the target discourse and the source discourse, we have consecutive interpreting (CI) versus simultaneous interpreting (SI), among others. The last dichotomy of interpreting performance, that of CI and SI, represents two dominant modes of interpreting, and they are the focus of our current study.

These two modes of interpreting performance entail different sub-level components. The composition and allocation of constituent mental operations during the process of interpreting are best captured by the Effort Model (Gile, 1995/2009, 2016). In this model, simultaneous interpreting (SI) is conceptualized as a process consisting of a listening and analysis effort, a short-term memory effort, a speech production effort, and a coordination effort, which can be mathematically visualized as L (Listening) + M (Memory) + P (Production) + C (Coordination). In contrast, Consecutive Interpreting (CI) is processed in two phases, namely, the comprehension phase (L + N note-taking + M + C) and the reformulation phase (R remember + Read note-reading + P + C; Gile, 1995/2009, 1999, 2016). In this way, Gile decomposes and identifies the behavioral stages of the SI and CI process, which sheds important light on interpreting pedagogy as it simulates the interpreting process. Such portrayals of CI and SI have provided inspiration for designing tailor-made instructions to help students and practitioners appreciate and understand the cognitive functions of the brain and yield practical tools for interpreting training. In addition, the identified behavioral stages of the SI and CI process contribute to locating and capturing the translanguaging spaces and moments. These are in turn replete with fluid and dynamic interactions of cognitive constructs, in joint interplay with the multimodal, multisemiotic, and multisensory resources employed by the interpreter during the successive “translanguaging moments” of meaning-making. Through observing such behaviors as pauses, gestures, facial expressions, and other small gestures, we are making inferences to conceive the emergence, attractor, and self-organization in an individual’s interpreting performance.

As SI and CI are assumed to entail distinct time sensitivity and may likely consume different amounts of cognitive resources (Timarová et al., 2011; Timarová, 2015), previous studies have been conducted in terms of inter-modal cognitive load in CI versus SI (Lv and Liang, 2019) via a product-oriented approach, and in terms of time lag (décalage), known as eye-voice span (mainly in SI) via a process-oriented approach. The latter has focused on the temporal delay between the source discourse input and the target discourse output to examine the cognitive loads in the interpreting process (Barik, 1973; Lee, 2002; Christoffels and De Groot, 2004; Kim, 2005; Čeňková et al., 2014). In light of the key tenets and principles of translanguaging theory (Li, 2018, 2022), the present study sets out to adopt the process-oriented approach, probing into the “‘spur-of-the-moment’ actions that are semiotically highly significant” (Li, 2011, p. 1222) to the interpreters, aiming to examine the momentary complexity and dynamic architecture of the interpreting process. To this end, we focused on the different workflow tasks of SI and CI to gain a better understanding of their underlying non-linearity, emergence, and self-organization dynamics in these two distinct modes of interpreting. We were hoping that through this micro-level perspective of translanguaging, the current understanding of interpreting engagement associated with the two distinct modes of interpreting could be further advanced.

## 3. The current study

This section reports on the details of the empirical study. It begins with the research questions and then moves on to discuss the background of the participants and the details of the research methods and procedures of the experiments.

### 3.1. Research questions

Inspired by translanguaging theory as our analytical lens, in this paper, we aim to answer the following questions:

1. How does the different time sensitivity of SI and CI result in different translanguaging performances/spaces?
2. In such translanguaging spaces of meaning-making moments during simultaneous interpreting, how do the cognitive constructs of SI interact within the interpreter’s full range of repertoire to adapt to its *stringent* time sensitivity and thus achieve optimal performance in interpreting?
3. By the same token, how do the cognitive constructs of CI interact within the interpreter’s full range of repertoire to adapt to its *delayed* time sensitivity and thus achieve optimal performance in interpreting?
4. What are the pedagogical implications of distinguishing the translanguaging spaces between SI and CI?

### 3.2. Participants

The participants in the present study were year two students of the Chinese-Portuguese Master of Translation and Interpreting students (*n* = 8) at a university in Macau. They were already familiar with interpreting after receiving one-semester training in consecutive interpreting in year one. When the data were collected, they were enrolled in two subject courses, namely, advanced interpreting and simultaneous interpreting. All students in the sample group passed the interpreting aptitude exam of Directorate General for Interpretation (DGI) before they studied interpreting. In this case, their language proficiency is at a similar level. Their subject teachers have confirmed their proficiency levels based on their performance thus far in the master’s program. We invited a total of eight students to participate in the study. They were all well informed and signed their consent to participate in the project (with the understanding that they could withdraw at anytime).

Among the eight students surveyed, three were men and five were women, with an average age of 24.5 years. Among them, four come from the Chinese mainland, and the rest are from Macau. Regarding the native language, three speak Mandarin as their native and dominant language in daily life, four speak Cantonese, and one speaks Portuguese. Most of them started learning Portuguese at university. It should be noted that the student whose mother tongue is Portuguese understands a little Cantonese and has been learning Mandarin since birth. All of the participants have passed the DGI Chinese-Portuguese/Portuguese-Chinese Interpreting Aptitude exam for a degree of Master in Chinese-Portuguese Translation and Interpreting, which ensures that all the participants have met the basic requirements for the professionalized training. Among them, three students have the certificate of the China Accreditation Test for Translators and Interpreters (Chinese-Portuguese) of level 3 (CATTI 3) and one with the certificate of level 2 (CATTI 2). Regarding proficiency in Portuguese as a foreign language, there is one student who has passed the Portuguese exam DUPLE (Diploma Universitário de Português Língua Estrangeira, equivalent to C2), four who have passed DAPLE (Diploma Avançado de Português Língua Estrangeira, equivalent to CI) and one who has passed Celpe-Bras – Avançado superior (Brazilian certificate in Portuguese as a foreign language, advanced superior level, equivalent to C2), which means they are proficient Portuguese users according to the Common European Framework of reference for Languages. In addition, two students attended and held English proficiency certificates, including China’s national Test for English Majors (TEM-8), Business English Certificate (BEC) Higher, and IELTS. All students had experience living or studying in Portugal, and two of them had been to Brazil. Most of them stayed there in the host country for 1 year and a half. During interpretation, seven students use Portuguese as Language B and the remaining one as Language A; three use Mandarin as Language A, three as Language B, and two as Language C; four use Cantonese as Language A and four as Language C; and three use English as Language B and five as Language C.

For Portuguese proficiency, the average self-assessment is 7.25 on a scale from 1 to 10, with the average of written comprehension being the highest (7.75) and written production being the lowest (6.75). Regarding Mandarin proficiency, the average is 8.68, with the average oral comprehension being the highest (9.13) and the written production the lowest (8.13). Concerning Cantonese proficiency, the average is 6.68, with the average for listening and writing the highest (7) and oral production the lowest (6.13). For Chinese-Portuguese interpretation proficiency, the self-assessment average is 5.66, with the highest average in the Mandarin-Portuguese combination (6.88) and the lowest in the Portuguese-Cantonese combination (4.5). For the Chinese-Portuguese translation proficiency, the self-assessment average is 6.84, with little difference between the various language combinations, and the highest average is in the Portuguese-Simplified Chinese combination (7) and the lowest in the Portuguese-Traditional Chinese and Portuguese-Simplified Chinese (6.75). Regarding their bilingual ability (Portuguese and Chinese), the average self-assessment was 6.75. Three of them have previous working experience as an interpreter.

### 3.3. Research methods and procedures

The empirical study was designed and conducted to answer the four research questions. Both a consecutive interpreting performance and a simultaneous interpreting performance of a group of students (from Portuguese to Chinese – Mandarin or Cantonese) were recorded, transcribed, and analyzed, followed by an inquiry of emotional engagement *a posteriori*. In addition to analyzing textual transcription as in most previous studies, we also analyzed multimodal transcription of the SI and CI interpreting data. We analyzed their note-taking – specifically for CI, facial expressions, gestures, images, and speech performance of interpreting. To triangulate these data, we conducted an emotional engagement survey to further probe into their emotions aroused in task interaction during interpreting, which can be enjoyment, boredom, tedium, discouragement, frustration, or annoyance (Aubrey, 2017; Phung, 2017; Dao and McDonough, 2018; Dao, 2019; Yoshida, 2020; Dao et al., 2021; Sampson and Yoshida, 2021).

Two interpreting tasks were selected for the students to complete: one was conducted as Consecutive Interpreting (CI) and the other as Simultaneous Interpreting (SI). Students were requested to finish the two tasks in the same sequence: CI first and then SI. Two recorded speeches were selected as the interpreting tasks from the DGI speech repository. Both tasks are of intermediate level (corresponding to the EU interpreting training criteria in terms of time duration, structure, complexity, vocabulary). Both tasks are in Portuguese, spoken by Portuguese native speakers. One task is for the SI and the other for the CI mode. Although the two selected speeches for interpreting were spoken by the same speaker, they were on two different topics unrelated to each other. The reason for choosing the speeches of the same speaker was to maintain the register, accent, and speech rate as stable variables to reduce the noise coming from the source language as much as possible. In addition, the mean sentence length, lexical variety (TTR) and lexical density of the two tasks are similar. More details of the two interpreting tasks are listed below in Table 1.

**Table 1:** Characteristics of the two interpreting tasks.

Speech details for the CI task	Speech details for the SI task
Speech number: 23904	Speech number: 23905
Duration: 04:37	Duration: 07:17
Language: (pt) portuguese	Language: (pt) portuguese
Level: Intermediate	Level: Intermediate
Use: consecutive	Use: simultaneous
Type: pedagogical material	Type: pedagogical material
Domains: Fisheries and Maritime Affairs, Humanitarian Aid	Domains: Energy, Environment
Mean sentence length: 29 words	Mean sentence length: 29 words
Type-token-ratio (TTR): 0.48	Type-token-ratio (TTR): 0.45
Lexical density: 22.92%	Lexical density: 29.4%

#### 3.3.1. Language directionality

Both tasks require interpreting performance from Portuguese to Mandarin/Cantonese. That is, the source discourse is in Portuguese, while the target discourse is in Mandarin or Cantonese.

#### 3.3.2. Test method

Before the test the students first conducted the Consecutive Interpreting task with notebooks provided to them beforehand. After CI, they performed the simultaneous interpreting task. In between the two exercises there was a break of several minutes for the participants to reduce the fatigue effect. The whole process was video-recorded. The recording took place in real-time, giving no chance of a second recording.

Due to the limited recording equipment and technical support, we divided the eight students into two groups. To avoid the exchange of information between the two groups, the experiment was arranged on the same day. All students were instructed to gather in the waiting room at the same time. Then, they were divided into two groups randomly. Group One went to the testing room first. Group Two stayed in the waiting room and entered the testing room after Group One finished the task.

#### 3.3.3. The setting of the equipment

In front of each student, there was a computer (screen) with two selected videos prepared in advance. Students used earphones to listen to the recorded video. The notebooks and the bilingual terminology sheets had been placed on the table before the experiment began. First, video A (for CI) was broadcast. After video A, students performed consecutive interpreting (which was video recorded). When the CI finished, video B (for SI) was broadcast, and students performed the simultaneous interpreting task.

#### 3.3.4. Recording procedures

For the CI task, the procedures included (1) the input stage— listening, memory, note-taking, and coordination; and (2) the output stage – remembering, note-reading, production, and coordination. Meanwhile, a camera was set and maintained at a 45° angle to capture the interaction between students and the (computer) screen, as well as the nonverbal reactions of students. Similarly, for the SI task, starting from the play of the source speech to the end of the whole performance, a camera was set and maintained at a 45° angle to capture the interaction between students and the screen as well as the nonverbal reactions of students.

#### 3.3.5. Materials prepared

All students were provided with notebooks, earphones, and bilingual terminology sheets. To ensure the smooth running of the experiments, we also adopted the following measures to reduce interferences: (1) Before the experiment, the students were briefed about the whole recording process, and (2) The student helpers (doctoral candidates in the translation/interpreting program) who recorded the video were trained not to distract the attention of students, and were instructed to remain silent throughout (having no interactions with the students during the whole recording process).

The detailed procedures of the experiments are summarized below:

1. The students were informed of the test in advance.
2. The test was conducted with their written consent signed in advance.
3. Before the tasks, all students were informed of the procedure and had half an hour of practice trials under the guidance of their interpreting teacher.
4. Notebooks for note-taking, pens, earphones, and terminology sheets were provided in the testing room.
5. When recording, each of the students was arranged in a separate classroom, with the camera already arranged in all four classrooms.
6. Before the experiment, research assistants double-checked to ensure that each camera operated normally.
7. The video was broadcast only after students were ready. Students controlled the start button and the ending button.
8. To guarantee the quality of the recording during the COVID-19 period, the mask could be temporarily removed, but social distance remained in force.
9. The test was not repeated. Participants had only one chance to do it.
10. After the experiment, all the notebooks were collected for data analysis.
11. After the recording, the technician checked whether the recording had worked well.
12. A Thank-You message was sent to all the participants after the whole procedure (via Wechat).

#### 3.3.6. Data transcription, alignment, and annotation

All 16 videos are subtitled using video editing software “Jianying” (Capcut in English). For the videos in Mandarin, the software has the function of automatically recognizing the voice and adding subtitles. As for the ones in Cantonese, subtitles were added manually by the authors. It is important to emphasize that the subtitles transcribe faithfully what the students were saying, including modal particles, pauses, repetition and grammatical errors. In the CI analysis, three episodes were selected from the CI speech for analysis. The related parts of the videos that present the participants’ interpreting multimodal and multisemiotic moments (including facial expressions and gestures) were captured in a screenshot and the corresponding parts of the notes taken town (including Chinese characters, words, letters, symbols and numbers) were also captured. In the SI analysis, two episodes were identified from the SI speech. The starting and ending times of all the pauses (over 5 s) in the videos that occur during the interpretation of these texts were registered and the corresponding parts of the videos that present the participants’ multimodal and multisemiotic interpreting moment (including facial expressions and gestures) were captured.

## 4. Data analysis

A total of 16 videos (CI and SI) and 8 notebooks were collected. The 16 videos were then transcribed, anonymized, and subtitled for analysis of meaning negotiation. The notebooks were digitalized, anonymized, and analyzed to understand the interpreting moments, especially for the CI task. Our current study focuses on the pauses shown in CI and in SI to examine the time-sensitivity of both modes of interpretation. Our goal is to probe into the underlying interplay of cognitive resources in the interpreting process implicated in these two distinct modes of interpreting. Inspired by translanguaging theory, we also analyzed multiple interpreting resources (including multimodal, multisemiotic and multilingual, and multisensory) such as note-taking (for CI) as well as non-verbal resources such as gestures, postures, and gaze (McNeill, 1992; Krauss and Hadar, 1999), to demystify the process of meaning-making and negotiation during interpreting.

For the video recordings, the multimodal transcription method was employed to analyze the SI and CI interpreting performance. The pause moments are captured in a screenshot and analyzed by referencing several transcription methods and conventions (c.f. Jefferson, 2004; Seedhouse and Richards, 2007; Mondada, 2018). For the CI task, the multimodal transcription was analyzed in a comparative manner: the pause screenshots were complemented by the note-taking pictures. This method was triangulated with an emotional engagement survey following the experiment. The analytical approach to emotions served to corroborate how the students perceive their own translanguaging practices at specific moments in interpreting performance, and this in-depth exploration of personal emotions helped investigate their performance. In our study, the moment analysis triangulated with the emotional survey helped to unveil the hidden competition among the cognitive constructs in the time-sensitive interpreting performance.

## 5. Results and discussion

The findings of the present study are presented in two sections that focus on the two interpreting modes, respectively. The first section provides an account of the translanguaging performance of the CI task and its time sensitivity observed in the episodes of the input stage and the output stage, during which note-taking and note-reading figure prominently, thus providing answers to research questions 1 and 2. The second section analyzes the translanguaging episodes of the SI task and its time sensitivity observed in the pause analysis, which complements research question 1 and answers research question 3, specifically. Taken together, these results and findings contribute to our deeper understanding of the meaning negotiation process during the interpreting process, ultimately illuminating research question 4.

### 5.1. Translanguaging moments and time sensitivity in CI

Compared with the SI task, the CI task did not have the same required time-sensitivity in terms of simultaneity. The two stages of the CI exercise, the input and output stages, render its time sensitivity focusing more on the interplay of working memory and note-taking and reading. Also, note-taking can be considered an external tool that helps to recall the original speech and thus becomes a technique or aid of memory, *per se* (Gillies, 2013). In interpreting, especially in consecutive interpreting, during which process an interpreter provides several rounds of interpretation, note-taking becomes an essential device to complement the limitation of short-term memory, an external device to strengthen our limited working memory capacity by reducing the cognitive load placed on the interpreting task (Cowan, 2000; Dong et al., 2018; Wen and Dong, 2019). In addition, in the CI exercise, the time sensitivity is visibly represented in note-taking, as during CI, the verbal inputs *via* mental exercises (comprehension, memory, analysis) are multimodally and semiotically transformed into visualized images, symbols, numbers, and linguistic representations in note-taking, which in turn helps to verbally transmit the voice in the output stage. The whole process of these “translanguaging spaces” is best captured and reflected in the notes taken by the interpreters. Following this rationale, we highlighted the note-taking of the participants (interpreters) of some critical moments to give them close snapshots.

To facilitate our analysis, we adopted multimodal transcription to analyze these momentary translanguaging spaces. Episodes of the original speech are selected from three aspects: (a) syntactically complex sentences,^1^ (b) semantically enriched sentences, and (c) sentences containing two or three figures. Notes taken by the participants were also screen captured to observe how information was visibly reflected. The interpreting output was also transcribed to triangulate the time sensitivity entailed in note-taking as a result of the competition of various cognitive loads (comprehension, memory, and note-taking). Sentences within each category were matched in terms of length and structure.

It should be noted that the interpreting textual transcript is exactly the same as what each student had uttered, including the modal particles (hesitations) and repetitions. In addition, we accompany the data with an image of the notes taken and a screenshot of the interpreting at the moment of interpreting to give a visual picture of the critical moments as important clues of the multi-dimensional and multimodality analysis.

#### 5.1.1. Multimodal transcription of CI exercise

The interpreting of the syntactically complex sentence involved one adverbial clause (to ensure…), one objective clause (European Union rules provide that…), and one attributive clause (fishery products that…). Thus, it constituted a demanding challenge for the cognitive load in comprehension and note-taking. Based on the eight students’ notes, the speech information was written down in various forms, including symbols, complete or simplified Portuguese words, abbreviations, and Chinese characters. All students used at least two of these forms to write the main idea of the original speech, resorting to their available repertoires in the cognitively demanding multitasking moment. As trained students, they only focused on the information instead of writing the complicated sentence structure. As to their notes, a diagonal layout is presented with some sufficient space (except for student F). However, due to this technique of note-taking, most students skipped the internal logic hidden in the syntactically complex sentence, resulting in piling-up information (especially in the cases of students E and G) and leading, to some extent, to their unsatisfactory performance at the output stage. Only students A and F managed to jot the word that indicates the logical relationship “para (to)” using its abbreviated form or in Chinese. Students A, B, and C used the symbol “$” to represent the original word “preço (price),” whereas others wrote the full expression of “preço.”

#### 5.1.2. CI moments of a semantically complex sentence

The students’ interpretation of the single sentence containing condensed information, including terminologies such as *preço de retirada comunitário* (community withdrawal price), *comissão* (commission), *peixe* (fish), *crustáceos* (crustaceans), and *mariscos* (shellfish). Therefore, it can be cognitively demanding to analyze, write, memorize, and process the information simultaneously.

The notes of the eight students came in different forms including symbols, abbreviations, complete and simplified Portuguese words, and Chinese characters. Although student G was weak in reproducing syntactically complex sentences with notes, she did well with the semantically complex sentences by relying on the list of terminologies.

#### 5.1.3. CI moments of a sentence with two figures

Regarding the CI moments of a sentence with two figures, this single sentence contains two figures, namely *mais de mil* (more than one thousand) and *mais de 200 mil* (more than 200 thousand). Attention should be allocated to the figures and the modifying components of “mais de” (more than). The notes of the eight students continued to be diversified. It was found that both Student E and G failed to write down any of these two figures in their notes, while student H wrote down one and all other students wrote down both.

#### 5.1.4. Summary of CI moments

In short, with the multimodal description of the CI moments of the eight students in interpreting three types of complex sentences from Portuguese to Chinese (Mandarin or Cantonese), we can discern the translanguaging spaces in note-taking and note-reading. Multimodal, multisemiotic, and multilingual signals were used in the note-taking stage (symbols, figures, abbreviations, words in Portuguese, Chinese, or English) when simultaneously, all the cognitive and sensory repertoire could be mobilized in listening, information analysis, memorization, and note -taking (input stage) and memory retrieval, note-reading, and production (output stage).

Furthermore, it could be observed from these moment analyses that the more diversified signals were used in note-taking, the better the coordination between comprehension, memory, notes, and production, and the better the interpretation performance. Those who were used to writing only one signal were accustomed to writing the words in their full expression, which was not a wise strategy in terms of time sensitivity. In addition in this complex process, attention allocation is another important intervening factor that can make a huge difference to the outcome of interpretation, as shown in the case presented above about interpreting figures. Many students neglected or forgot the modifying components of the figures in their notes, which influenced their memory retention and consequently affected the final performance. On the other hand, in the production phase when there was difficulty in retrieving the contents from memory or reading the notes, students usually uttered broken language and made facial expressions or body gestures. Specifically, they uttered modal particles, unduly repeated words, with temporal stuttering, long pauses, or a slowed-down pace. Sometimes. they made gestures, frowned, repeatedly looked around, and played with a pen in hand.

Regarding the accuracy and integrity of the production of the three types of complex sentences, the students encountered greater difficulty in processing, noting, and interpreting the information of the syntactically complex sentence than in the semantically complex sentence and the sentence with two figures. This is mainly due to the large difference between Portuguese and Chinese syntax, which requires extra cognitive effort in segmentation and analysis in our mental space. This implies that more attention could be paid to the practice of interpreting syntactically complex sentences during interpreter training in the future.

### 5.2. Translanguaging moments and time sensitivity in SI

In the simultaneous interpreting (SI) task, the time-sensitivity is entailed in the competition of the entire cognitive load implicated, namely when the input verbal information is visualized and archived in the mental space, processed simultaneously with newly input verbal information, and then verbally rendered in another language. As shown by many SI studies, the whole process tolerates a time lag of between 2 and 5 s; otherwise, the accuracy is considerably endangered (Barik, 1973; Lee, 2002). The time lag can serve as a temporal variable and sensitive measure to reflect the speed of underlying processing in the cognitive approach to research on interpreting (Timarová et al., 2011; Timarová, 2015). Pause detection and analyses have been studied in language and translation production (Butterworth, 1980; Schilperoord, 1996; Jakobsen, 1998; Dragsted and Hansen, 2009). In the past 20 years, pauses have been investigated as a measure of (dis)fluency in interpreting studies (Tissi, 2000; Pio, 2003; Tohyama and Matsubara, 2006; Xu, 2010; Han, 2015; Yang, 2015; Song and Cheung, 2019; Han and An, 2021). Though different research studies have implemented different lengths, the thresholds of pause have varied from the low cut-off points of 0.25 s to the high cut-off points of 5 s. To gain a deep insight into these pauses, we have opted to select the highest end of cut off points, i.e., 5 s in the present study. As an observable variable, a pause can visibly show the hesitation and boundaries between the verbal input and verbal output in SI and make the complex moments of competition of cognitive load visualized semiotically. With this rationale, we applied pause analysis as a measure of time sensitivity in analyzing the SI task and focused on the pauses in this study that lasted longer than 5 s. As the statistics show (in the following table), in the CI exercise, no pauses of over 5 s were detected in this trained group, while in the SI exercise, almost all the participants showed some pauses in their performance (between 2 and 18 times), except for one participant who had worked as a professional interpreter before enrollment in the course. The following table shows the pause situation in the performance of the CI and SI tasks in terms of time sensitivity (Table 2).

**Table 2:** Pauses in the performance of the CI versus the SI task.

Participants (Pseudo names)	Time of note-taking	Time of CI	Time of SI	Pauses in CI (longer than 5 s)	Pauses in SI (longer than 5 s)
A	4 min 35s (275 s)	4 min 01s (241 s)	7 min 23s (443 s)	No	No
B	4 min 31s (271 s)	4 min 16s (256 s)	7 min 18s (438 s)	No	1) 06:29–06:39; 2) 07:34–07:40
C	4 min 19s (259 s)	3 min 20s (200 s)	7 min 18s (438 s)	No	1) 01:24–01:34; 2) 01:39–01:51; 3) 02:01–02:12; 4) 02:17–02:23; 5) 02:29–02:38; 6) 02:47–02:54; 7) 03:18–03:32; 8) 03:43–03:52; 9) 04:27–04:33; 10) 04:52–05:07; 11) 05:24–05:32; 12) 06:00–06:17; 13) 07:05–07:22; 14) 07:31–07:43
D	4 min 50s (290 s)	3 min 42s (222 s)	7 min 14s (434 s)	No	1) 03:24–03:33; 2) 04:37–04:45; 3) 04:49–04:55; 4) 05:01–05:08; 5) 05:29–05:38; 6) 05:51–05:57; 7) 06:46–06:55; 8) 07:14–07:20; 9) 07:29–07:38; 10) 08:02–08:11; 11) 08:26–08:34
E	5 min 08s (308 s)	5 min 59s (359 s)	7 min 20s (440 s)	16:50–17:00	1) 06:35–06:41; 2) 06:47–06:56; 3) 09:30–09:36; 4) 11:09–11:17; 5) 12:05–12:11
F	5 min 24s (324 s)	5 min 17s (317 s)	7 min 26s (446 s)	No	1) 02:40–02:50; 2) 03:36–03:44; 3) 04:35–04:46; 4) 05:58–06:06; 5) 06:23–06:31; 6) 06:50–06:59; 7) 07:20–07:27
G	4 min 43s (283 s)	3 min 39s (219 s)	7 min 18s (438 s)	06:43–06:49	1) 00:51–01:02; 2) 01:33–01:48; 3) 02:07–02:18; 4) 02:34–02:41; 5) 02:49–03:06; 6) 03:14–03:27; 7) 03:37–03:55; 8) 03:56–04:07; 9) 04:13–04:22; 10) 04:26–04:36; 11) 04:56–05:06; 12) 05:30–05:41; 13) 05:48–05:57; 14) 06:03–06:14; 15) 06:21–06:34; 16) 06:35–06:41; 17) 06:56–07:28; 18) 07:29–07:35
H	6 min 34s (394 s)	3 min 28s (208 s)	7 min 19s (439 s)	No	1) 03:45–03:51; 2) 04:23–04:29; 3) 04:41–04:48; 4) 05:15–05:24; 5) 05:48–05:55; 6) 06:08–06:17; 7) 06:43–06:51; 8) 07:57–08:08; 9) 08:32–08:39; 10) 09:21–09:27
Mean	≈5 min (300.5 s)	≈4 min12s (252.8 s)	≈7 min18s (439.5 s)		
SD	≈43.1	≈56.6	≈3.6		

In the following analysis, we will only focus on the pauses detected in the SI performances that lasted longer than 5 s, with a view to probing the internal cognitive struggle and its external manifestations. The multisemiotic, multimodal, and multisensory manifestations (gestures, facial expressions, gazes, etc.) of participants are examined at the pause moments, along with the performance information (such as accuracy, loss, omission) to capture the translanguaging space in the complex moments of SI.

Again, the multimodal transcription method was adopted to present the pause analysis in the SI task. Episodes of the pauses that the participants had in common are highlighted for detailed analysis. The input sentences were located and selected following the identified pauses. The pause moments (the first 5 s) of participants were screen-captured to see how the participants reacted to the pause. As one participant did not show any pauses, his or her interpreting output is presented as a benchmark to contrast the pause phenomenon of the other participants to detect the different cognitive manipulation in favor of time sensitivity in SI.

In the analysis of the SI task, the most common pauses among the eight students are presented. The utterances with which the vast majority of students presented a long pause during interpretation, along with the location of the respective pauses in the recorded videos, the interpretation transcripts, and the screenshots. We found that the focused pause occurred in a syntactically and semantically complex sentence. It is an appositive clause, containing an adverbial clause of result (since…), with a gerund clause (increasing…). In the sentence, the expression of the transition phrase “not even (not even)” has a meaning contrary to what was said earlier, thus increasing the complexity. Furthermore, the contents of the sentence were less common in everyday life. Additionally, the verb tenses were varied (present tense, infinitive, present subjunctive). During the interpretation, all students except for student A had one to three long pauses (longer than 5 s). In Student B’s pause (which lasted 6 s), the student stared at the screen, looking around at times, with a struggling facial expression and frown. The information she reproduced, although informational, was contrary to the meaning of the original discourse. Student C had a longer pause (approximately 17 s), stared at the computer, and looked around from time to time. She did not understand the original speech, and the information she reproduced was merely the repetition of the previous information in other sentences. Regarding Student D’s pause (of 9 s), the student stared at the computer, with a frown on her face and compressed lips. She followed the principle of “listening to one’s self while speaking” during SI, leaving one ear untapped by earphones. In production, she correctly reproduced part of the speech but missed the logical linking words [that is, the word “nem sequer” (not even)]. At Student E’s pause (of 6 s), the student stared at the computer, with one hand covering the headset. At student F’s pause (of 9 s), the student stared at the computer with one hand supporting his head. He also followed the principle of “listening to one’s self while speaking,” leaving one ear untapped by earphones. During the pause, he frowned, touched his nose, showing the inner struggle of not understanding the original speech and producing irrelevant speech. The content produced did not correspond to the original meaning. Student G had two subsequent long pauses. In the first pause (practically 32 s), the student stared at the screen (the speaker), adjusted the headset, and moved her lips, with a hand supporting the chin. After uttering a modal particle (hesitation), there came another pause (of 6 s), during which time the student continued to stare at the screen with one hand on the headset, frowning. The production revealed that she did not understand the original content and produced nothing. Regarding student H’s pause (of 6 s), the student blinked continuously, with one hand pressing all the time on the handset. He also moved his body and lips, which manifested his physical discomfort aroused by cognitive hardship in understanding the speech.

In short, by using the selected sentence with a complex syntactic structure and rich meanings, even the trained students showed their inner mental struggle by manifesting diversifying reactions in response to the cognitive torture. This situation also corresponds to what we have found in the case of the CI task. Considering that SI requires stronger time sensitivity than the CI task, we found that gestures and facial expressions during SI were less frequent and less diverse than those in CI, given that the students needed to look at the screen (by gazing) to pay close attention to the speaker and the speech (by pressing the earphone set), rather than looking primarily at the note written during CI (by glancing at glossary, looking around, pen-playing and gesturing). Furthermore, during SI, when hearing information that they cannot understand, students tend to omit that information to avoid missing the following information. In the case of the CI task, on the contrary, when students cannot understand or reproduce what they hear and what they have written, they also resort to reimagination to complete the original information instead of omitting that information, which is reflected in pen-playing, looking around, although all these gestures and facial expressions happen unconsciously.

### 5.3. Emotional engagement survey

To triangulate the SI and CI interpreting data, we also conducted an emotional engagement survey. As both the SI and CI reflected the inner mental struggle of students, such a survey of their emotional engagement could be revealing. The survey was composed of 10 descriptions, of which the first five were positive and the remaining five were negative. Students were requested to assess their actual situation according to these descriptions on a scale of 1 to 10 (1 refers to “strongly disagree” and 10 to “strongly agree”). The results and findings of the survey are presented in the following Table 3.

**Table 3:** Results of the emotional engagement.

Number	Description	Average	SD
1	I felt that the task was enjoyable to do.	6.50	1.51
2	I felt interested while I was doing the task.	6.63	1.06
3	I felt excited while I was doing the task.	6.00	2.00
4	I felt content while I was doing the task.	5.25	1.49
5	I felt satisfied while I was doing the task.	4.50	1.69
Mean of 1–5		5.776	
6	I felt bored while I was doing the task.	8.38	2.26
7	I felt the task was tedious.	8.63	1.77
8	I felt annoyed while I was doing the task.	7.75	1.67
9	I felt discouraged while I was doing the task.	6.63	2.67
10	I felt frustrated while I was doing the task.	6.50	2.62
Mean of 6–10		7.578	

Based on the answers collected, the average and the standard deviation (*SD*) of each description presented above were calculated. From the table, it can be observed that the scores attributed to students’ responses to the five negative-sense descriptions are higher (mean of 7.579) than those to the five positive-sense descriptions (mean of 5.776). That is, all of the respondents affirmed that the tasks were not tedious and that they did not feel annoyed during the tasks. Furthermore, most respondents indicated that the tasks were enjoyable and made them feel interested. However, generally speaking, students did not feel satisfied with their performance while performing the two interpreting tasks. This may be related to the fact that their translation and interpretation proficiency was still not very advanced, considering the low average self-assessment in the background survey. Even so, they did not get frustrated. Therefore, it can be concluded that the students had a moderately positive attitude toward the two tasks of SI and CI exercise.

## 6. Conclusion and pedagogical implications

Based on the different time-sensitivity of CI and SI, this present paper examines the momentary complexity and dynamic architecture of the distinct workflow tasks of the two modes of interpreting within the working definition of translanguaging and its key tenets. We focused on interpreting engagement from a micro-level perspective as inspired by the method of “moment analysis” advocated by the emerging translanguaging theory (Li, 2011, 2018, 2022). For the CI task, as note-taking was the nexus connecting the input and output stages, the translanguaging space of note-taking was analyzed to better understand the underlying non-linearity, emergence, and self-organization dynamics of listening comprehension and production. For the SI task, pauses (of over 5 s) were selected as the departure point, as pauses are sensitive to the ear-voice span between the hearing of the source speech and its corresponding reformulation. The intense struggle of the cognitive process of SI is manifested in the translanguaging space of pauses. In addition to textual transcription, we also employed multimodal transcription to analyze the SI and CI interpreting data, which were further triangulated by a follow-up emotional engagement survey.

The empirical study of analyzing the CI and SI momentary performances allows us to identify the segments that are cognitively demanding in interpreting performance. For the CI task, three types of segments were selected according to their complexity in terms of syntax, semantics, and quantity of figures. It turned out that syntactically complex sentences constituted the most difficult task even for trained students, while semantically complex sentences or sentences with a certain quantity of figures are relatively less difficult. The more demanding the task, the more hesitations with modal particles were shown and complemented with gestures, facial expressions, and uneasy body adjustment as well as mobilization of other extralinguistic repertoires. However, if the notes were diversifying and well organized (especially those with logical links), fewer hesitations were detected. The analysis of the translanguaging space in the notes thus helps us to organize the working brain and keep the workflow in order.

In contrast, SI performance occurs during a much shorter time lag between understanding and reproduction. In the pause analysis of the SI task, it was found that almost all the pauses occurred in syntactically and semantically complex sentences, which again confirmed the patterns detected in the CI task. The misunderstanding and infelicities such as omission, incompleteness, and incoherence all occurred in the long pauses (of over 5 s). All other sensory efforts such as gaze, frowning, and ear pressing that happened frequently attests to the inner cognitive struggle, which, in turn, shows competition in mental space and attentional control.

Taken as a whole, the results and findings of the empirical study reported here further corroborate that simultaneous interpreting and consecutive interpreting entail different levels of time sensitivity and likely consume different amounts of cognitive resources within the translanguaging environments. As such, it is hoped that the present study serves as a good starting point to find better solutions in interpreting pedagogy that will orient the interpreting students’ coping strategies in attentional allocation and control, both in CI and in SI. The field is expecting more research into this line of inquiry.

## Data availability statement

The original contributions presented in the study are included in the article/supplementary material, further inquiries can be directed to the corresponding author.

## Author contributions

LH and ZW: conceptualization, methodology, and writing—review and editing. JL: data collection. JL and YT: data analysis. LH, JL, and YT: writing—original draft preparation. LH: funding acquisition. All authors contributed to the article and approved the submitted version.

## Funding

The work was supported by a Macao Polytechnic University Research Project (RP/FLT-11/2022).

## Conflict of interest

The authors declare that the research was conducted in the absence of any commercial or financial relationships that could be construed as a potential conflict of interest.

## Publisher’s note

All claims expressed in this article are solely those of the authors and do not necessarily represent those of their affiliated organizations, or those of the publisher, the editors and the reviewers. Any product that may be evaluated in this article, or claim that may be made by its manufacturer, is not guaranteed or endorsed by the publisher.

## Footnotes

^1^To guarantee the objective measure of the syntactically complex sentences, we adopted the Index of Syntactic Complexity (Beaman, 1984; Givón, 1991) which emphasizes the subordinate clause structures and embeddedness.
